# Acute retroviral syndrome mimincs dengue in Singapore, a dengue endemic country

**DOI:** 10.1186/1742-4690-9-S1-P72

**Published:** 2012-05-25

**Authors:** JH Tan, A Verrall, S Archuleta

**Affiliations:** 1 Infectious Diseases at National University Singapore, Singapore, Singapore

## Introduction

The differential diagnosis for dengue-like illnesses is broad. However, World Health Organization (WHO) recommended confirmatory testing may not always be feasible. Acute human immunodeficiency virus type-1 (HIV-1) presents similarly to dengue and is an important diagnosis that may be missed when dengue confirmatory testing is not performed.

## Methods

Cases of acute HIV-1 in adults >15 years diagnosed between January 2010 and December 2011 were identified through HIV service records at our institution. Medical records were reviewed for admitting diagnosis, clinical and laboratory features.

## Results

Eight patients with acute HIV-1 were identified in the study period, seven of whom were clinically diagnosed as dengue and met the WHO dengue case definition. The duration of illness and clinical features were indistinguishable from dengue: fever, myalgias, nausea and rashes. Five had thrombocytopenia and three had leukopenia. During the same period, emergency physicians at our hospital assigned a dengue diagnosis using prespecified ICD-9 based coding for 276 persons over 15 years old. The ratio of acute HIV-1 cases to those with presumed dengue was 0.025.

## Conclusion

Acute HIV-1 infection can mimic symptoms of dengue fever. This case series adds to the single case report in the published literature. The ratio of 0.025 is concerning as not all patients with presumed dengue undergo confirmatory testing. This high rate of acute HIV-1 among dengue-like presentations needs further prospective investigation. Physicians in endemic areas like Singapore should test for acute HIV-1 in cases of presumed dengue where dengue confirmatory testing is negative.

